# Contrast-enhanced ultrasound findings of sclerotic nodules in Wilson disease: A case report

**DOI:** 10.1097/MD.0000000000040018

**Published:** 2024-10-18

**Authors:** Cong Hu, Kun Liu, Aihua Liu, Weiling Huang, Zhiwei Zhao, Yuancheng Jiang, Yixin Chen, Qi Zhang, Ying Wang, Meng Wu

**Affiliations:** a Department of Ultrasound, Zhongnan Hospital of Wuhan University, Wuhan, Hubei, China; b Department of Ultrasound, Third People’s Hospital of Hubei Province, Jianghan University, Wuhan, China; c Department of Ultrasound, The Sixth Hospital of Wuhan, Affiliated Hospital of Jianghan University, Wuhan, China; d Department of Pediatrics, Xianning Central Hospital, The First Affiliated Hospital of Hubei University of Science and Technology, Xianning, China; e Department of Radiology, Xianning Central Hospital, The First Affiliated Hospital of Hubei University of Science and Technology, Xianning, China; f Teaching Office, Zhongnan Hospital of Wuhan University, Wuhan, Hubei, China.

**Keywords:** contrast-enhanced ultrasound, diagnosis, liver cirrhosis nodules, slightly higher enhancement, Wilson disease

## Abstract

**Rationale:**

Wilson disease is an autosomal recessive genetic disease found by Samuel Alexander Kinnier Wilson and prevalent in childhood and adolescents.

**Patient concerns:**

An 18-year-old female patient presented to our hospital with a continuous decrease of 3 blood cell lines for more than 10 days, and diagnosed as decompensated cirrhosis. Ultrasonography showed diffuse lesions in the hepatic parenchyma, with multiple hypoechoic light masses in the parenchyma, the outline was still clear, and the internal echo was uneven. Contrast-enhanced ultrasound showed that the nodules were enhanced rapidly and uniformly, with an initial enhancement time of 9 seconds and a peak time of 17.2 seconds. The washing time was slightly earlier than that of the hepatic parenchyma and showed slightly higher enhancement in the delayed phase. Finally, ultrasound-guided biopsies showed unexplained liver cirrhosis.

**Diagnoses:**

Combined with clinical examination, it was inferred to be Wilson disease. It is difficult to diagnose hepatolenticular degeneration because of its concealed incidence, complex clinical manifestations, expensive detection of the *ATP7B* gene, and lack of other specific imaging signs.

**Outcomes:**

After admission, the patient was given symptomatic support treatment such as liver protection.

**Interventions:**

The patient was discharged after improvement of symptoms.

**Lessons:**

Here, the results of contrast-enhanced ultrasound in our case may provide a new idea for the diagnosis of Wilson.

## 1. Introduction

Wilson disease, also known as hepatolenticular degeneration, is an autosomal recessive genetic disease found by Samuel Alexander Kinnier Wilson and prevalent in childhood and adolescents.^[1,2]^ Its clinical manifestations are complex and diverse, with liver damage, neuropsychiatric dysfunction, Kayser-Fleischer (K-F) rings, and kidney damage as the main manifestations.^[3]^ After reasonable treatment, the prognosis of Wilson disease is good, but it is often delayed due to untimely diagnosis, resulting in a poor prognosis.^[4]^ Here, through this case report, we found that contrast-enhanced ultrasound (CEUS) has a certain value in the diagnosis of this kind of disease, suggesting that this imaging manifestation is likely to become the theoretical basis for the diagnosis of Wilson disease.

## 2. Case presentation

An 18-year-old female patient was admitted to the Department of Hepatobiliary surgery of our hospital because she found that the number of 3 blood cells decreased for >10 days. When she was admitted to the hospital, she mainly carried a summary of the discharge from the people’s Hospital of Qichun. After careful physical examination, it was found that the hemogram of the patient was slightly poor, the skin was not yellow, the liver and palm was mild, the transverse finger under the liver ribs was 2 fingers, the spleen was enlarged, the boundary was located at the navel level, there was no percussion pain in the liver and spleen area, and the mobility dullness was negative. In the past, she underwent appendicitis surgery at the age of 8, had a history of thrombocytopenic purpura, denied a history of hepatitis B, and drinking. Based on the above, the admission diagnosis was diagnosed as decompensation stage of liver cirrhosis.

**Figure 1.**
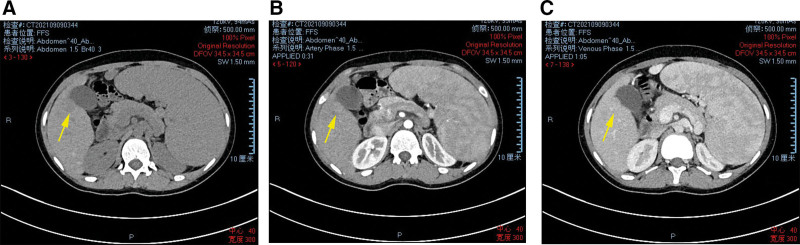
Plain scan and enhanced CT results. The results of plain scan showed that there were many slightly high-density shadows in the hepatic parenchyma (A), and the results of contrast-enhanced scan showed slight enhancement in arterial phase (B) and slight regression in portal phase (C).

**Figure 2.**
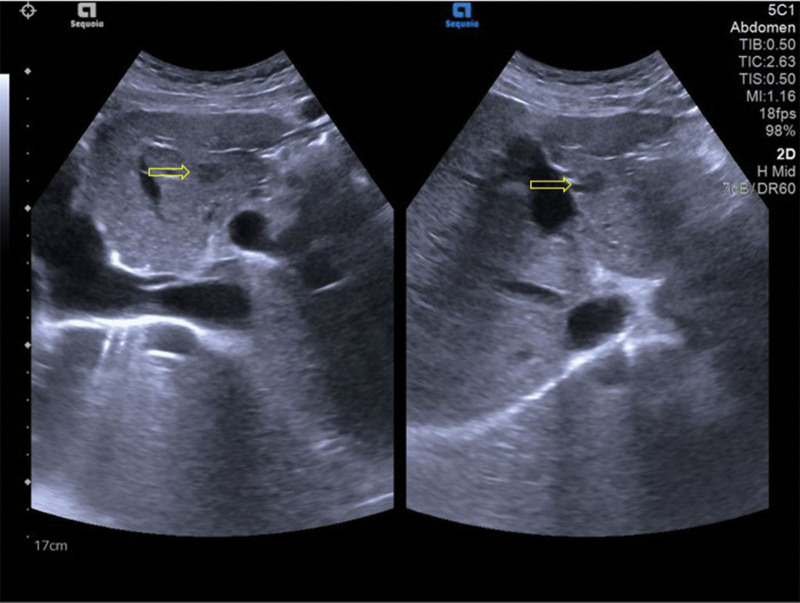
The results of two-dimensional ultrasound examination of the liver. The shape and section of the liver were normal, the edge of the liver was smooth, the light spots in the liver were thickened and enhanced, the distribution was uneven, and the blood vessels are clear. Multiple hypoechoic nodules can be seen in the liver (indicated by the yellow arrow), with clear contours and uneven internal echoes.

**Figure 3.**
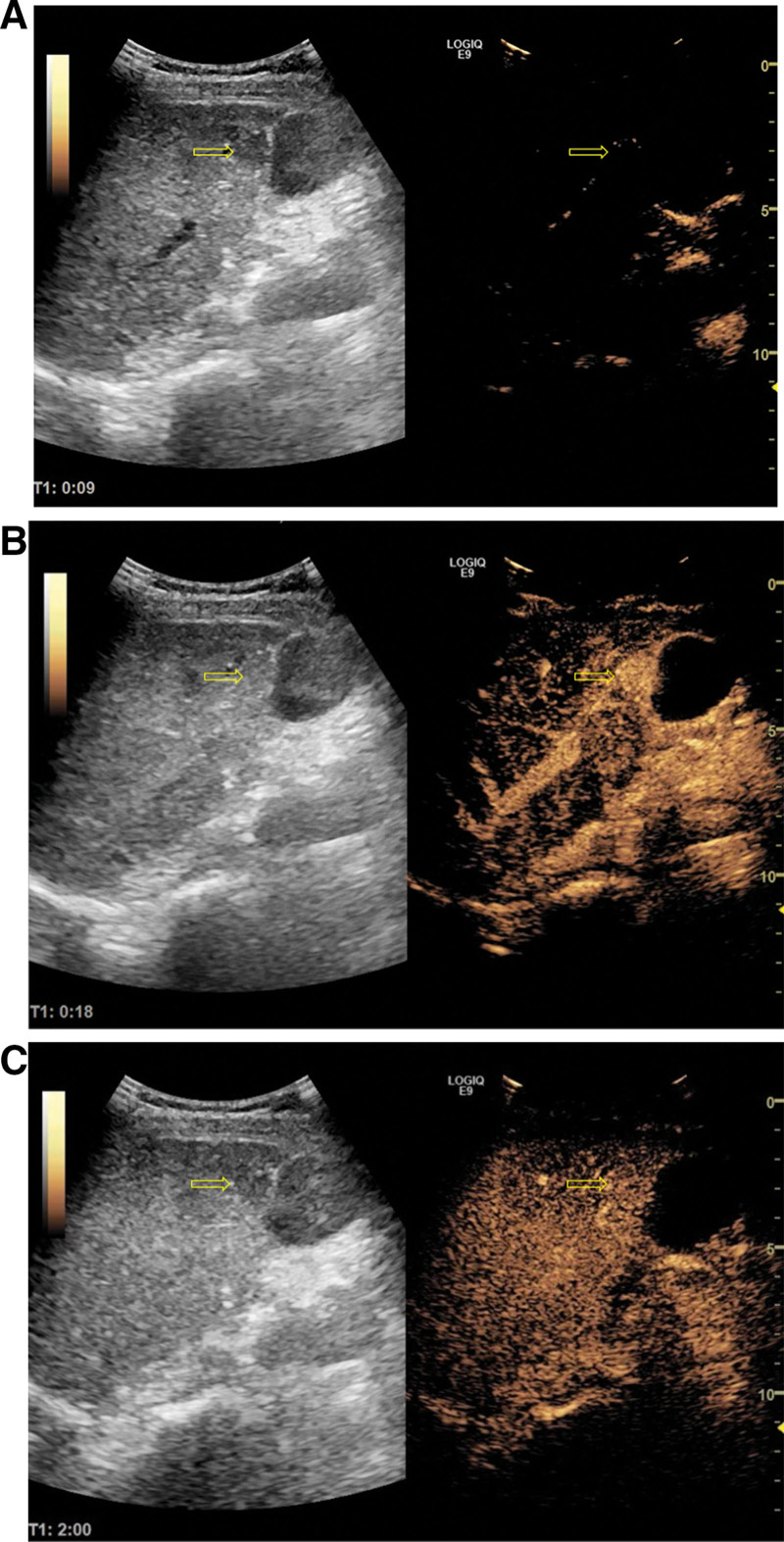
The results of CEUS in detection of hepatic nodules. (A) The results of arterial phase were examined by CEUS. (B) The arterial phase results of CEUS showed that the enhancement of hypoechoic nodules reached the peak at 17.2 seconds, which was much stronger than that of the hepatic parenchyma. (C) The results of CEUS showed that the hypoechoic nodules showed slightly higher enhancement in the delayed phase. CEUS = contrast-enhanced ultrasound.

**Figure 4.**
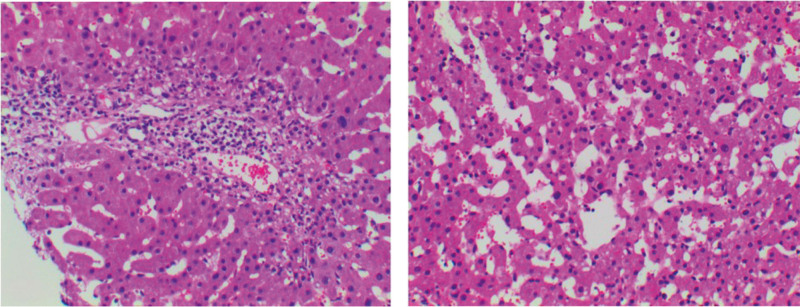
Pathological results of liver biopsy. The normal liver structure was replaced by bridged fibrotic scars and regenerated nodules of different sizes. The proliferation of fibrous tissue in the portal area and part of the hepatocytes was accompanied by lymphocyte and neutrophil infiltration, and local interfacial inflammation could be seen. Dilatation of hepatic sinusoids. Steatosis of some hepatocytes and no obvious cholestasis were found. Masson trichrome and reticular fiber staining showed liver fibrosis, D-PAS staining showed Kuffer cells, Prussian blue staining was negative. It is suggested that it is cryptogenic liver cirrhosis.

After admission, patients were given blood samples to detect liver function (ALT 62 U/L, AST 63U/L), blood routine and tumor markers. The results showed that liver function was impaired, white blood cells (WBC), red blood cells, hemoglobin (98.9 g/L), and platelets were lower than normal, and CA125 were increased. At present, the cause of liver cirrhosis is unclear. The whole abdomen CT results showed that there were multiple slightly high-density nodules in the liver parenchyma, and liver cirrhosis and regenerative nodules were considered (Fig. 1). The results of liver ultrasound examination at the same time showed that the liver parenchyma showed diffuse lesions, multiple hypoechoic light masses could be seen in the liver parenchyma, the outline was still clear, the internal echo was not uniform, and the space occupying effect was obvious (Fig. 2). In further CEUS examination, SonoVue 2.4 mL was injected into the median vein of the left upper limb, and the low echo light mass of the liver was enhanced rapidly and uniformly. The beginning time of enhancement was 9.0 seconds (Fig. 3A), which was earlier than that of the liver parenchyma, and the peak time was 17.2 seconds (Fig. 3B). The washout time was slightly earlier than that of the liver parenchyma, and showed continuous slightly higher enhancement in the delayed phases (Fig. 3C). The patient’s liver condition was recorded using ultrasonography to capture real-time images of the liver (Video S1, Supplemental Digital Content, http://links.lww.com/MD/N738). The patient performed liver biopsy under the guidance of ultrasound (Fig. S1, Supplemental Digital Content, http://links.lww.com/MD/N739), and the pathological results showed cryptogenic liver cirrhosis. combined with clinical examination (negative hepatitis markers and autoimmune related tests), it was inferred that chronic hepatitis with liver cirrhosis of unknown cause could be consistent with Wilson disease (Fig. 4), which needed to be further diagnosed by serum ceruloplasmin (CER) or free copper and genetic testing. The serum CER was detected in the patients, and the result was 0.16 g/L, lower than the normal value. At the same time, an ophthalmologist was invited to consult with an expert, and the results showed that the patient did not have K-F rings. In addition, the patient’s family refused genetic testing. Based on the patient’s examination, the diagnostic score can be calculated as follows: neuropsychiatric symptoms: the patient presented with sleep disorders, insomnia, irritability, and emotional lability, which were indicative of neuropsychiatric manifestations associated with Wilson disease. This category was assigned 2 points. Coombs negative hemolytic anemia: The patient’s hemoglobin level was 98.9 g/L, which may suggest the presence of hemolytic anemi when negative for the Coombs test, got 1 point. Serum CER: the serum CER level was 0.16 g/L (16 mg/dL), which was significantly lower than the normal range and was a typical finding in Wilson disease. This category was assigned 1 point. We scored the patient with a total of 4 points on the Leipzig score, which typically indicates that the patient meets the diagnostic criteria for Wilson disease. In summary, the patient was considered to be diagnosed with liver cirrhosis caused by Wilson disease. After admission, the patient was given symptomatic support treatment such as zinc salts (150 mg/d), low copper diet, liver protection, and discharged after improvement of symptoms.

## 3. Discussion

In our patients, the diagnosis of Wilson disease was made by liver biopsy combined with CER. The concealment of onset and the nonspecific findings of imaging examination make the diagnosis more difficult. According to statistics, the incidence of Wilson is 1/30,000.^[5]^ Wilson disease is mainly affected by gene mutation and environmental factors. The mutated genes mainly include ATP7B mutation in chromosome 13 or genes related to triglyceride metabolism, fat metabolism, and antioxidant pathway.^[6,7]^ The environmental factors include gender, iron, and diet.^[8,9]^

The clinical manifestations of Wilson disease are caused by the accumulation of serum free copper, resulting in nervous system symptoms, liver damage, and K-F ring changes. It is worth noting that previous studies have shown that patients with liver as the main manifestation tend to be younger than patients with nervous system changes,^[10]^ while K-F ring does not appear in all Wilson patients,^[11]^ only in 30% to 50% of patients with liver lesions, which are consistent with our case reports. Liver lesions caused by Wilson disease often progress from initial mild liver function changes to chronic active hepatitis, then cause liver fibrosis, and eventually develop into decompensated cirrhosis.^[12]^ Although studies have shown that patients with Wilson disease have a lower risk of liver cancer, liver cirrhosis increases the risk of death.^[13,14]^ Therefore, early diagnosis is undoubtedly the focus of improving the prognosis of Wilson disease.

Because of the rarity of the disease, Wilson disease usually considered after a long period of exclusive diagnosis. The gold standard for the diagnosis of Wilson disease is ATP7B gene testing,^[4]^ but it is often abandoned because of limited economic conditions. Secondly, the decrease of serum CER, the increase of serum free copper, liver function damage, hemolytic anemia, thrombocytopenia, and brain MRI are auxiliary diagnostic basis.^[4]^ Our case also echoes some of the above diagnostic basis.

At present, the imaging diagnosis of liver damage in Wilson disease has not been reported. In our case report, ultrasound technology was fully used to assist in the diagnosis. Two-dimensional ultrasound diagnosis showed that the patient’s liver diffuse venereal disease with multiple space-occupying nodules. Considering that the accuracy of traditional ultrasound in distinguishing benign and malignant lesions is only 23% to 68%, while the accuracy of CEUS is 92% to 95%.^[15]^ We suggest that the patients should be examined by CEUS, and the results suggest that there is a great possibility of benign space-occupying lesions in the liver. In addition, in the delayed phase, it is different from the complete non-enhancement of malignant lesions,^[16,17]^ the complete high enhancement of hemangioma,^[18]^ the non-enhancement of central scar of focal nodular hyperplasia,^[16,17]^ and the mild low enhancement of hepatocellular adenoma,^[15,19]^ It is also different from the isoechoic changes of liver cirrhosis compared with normal liver tissue in arterial phase and delayed phase,^[20]^ the result of our case is rapid enhancement in arterial phase and a slightly higher persistent enhancement in the delayed phase. Most importantly, the biopsy results of hypoechoic nodules in the liver are consistent with Wilson disease. Therefore, we can infer that in the process of CEUS, the contrast medium is rapidly enhanced, the rapid washout after reaching the peak, and the delayed phase shows persistent high enhancement, which is likely to become a suggestive basis for the diagnosis of Wilson disease. Of course, this sign still needs to be supported by a large number of clinical cases.

In conclusion, a high degree of suspicion index is required for the diagnosis of Wilson disease. As the onset of Wilson is not specific, incorrect treatment will delay the disease and lead to poor outcome, on the contrary, causal treatment can quickly improve the prognosis. Therefore, it is recommended that all cirrhotic nodules should be examined by contrast-enhanced ultrasonography. Hypoechoic nodules caused by unexplained liver cirrhosis are rapidly developed and cleared by SonoVue in the process of CEUS, accompanied by slightly higher persistent enhancement in the delayed phase. According to the imaging findings, it can be considered that hepatolenticular nodules may be caused by Wilson disease, of course, accurate diagnosis needs to be combined with other clinical indicators.

## Author contributions

**Conceptualization:** Cong Hu, Weiling Huang, Yixin Chen, Ying Wang, Meng Wu.

**Data curation:** Kun Liu, Aihua Liu, Meng Wu.

**Formal analysis:** Meng Wu.

**Funding acquisition:** Meng Wu.

**Investigation:** Zhiwei Zhao, Qi Zhang, Ying Wang, Meng Wu.

**Methodology:** Weiling Huang, Zhiwei Zhao, Yixin Chen, Ying Wang, Meng Wu.

**Project administration:** Meng Wu.

**Resources:** Meng Wu.

**Software:** Aihua Liu, Yuancheng Jiang, Meng Wu.

**Supervision:** Weiling Huang, Meng Wu.

**Validation:** Meng Wu.

**Visualization:** Meng Wu.

**Writing – original draft:** Cong Hu, Ying Wang, Meng Wu.

**Writing – review & editing:** Cong Hu, Ying Wang, Meng Wu.

## Supplementary Material


