# Supplementary figures and images for: Contrast-enhanced ultrasound findings of sclerotic nodules in Wilson disease: A case report
Source: Medicine (Baltimore). 2024 Oct 18;103(42):e40018. doi: 10.1097/MD.0000000000040018 (PMC11495761; doi:10.1097/MD.0000000000040018)

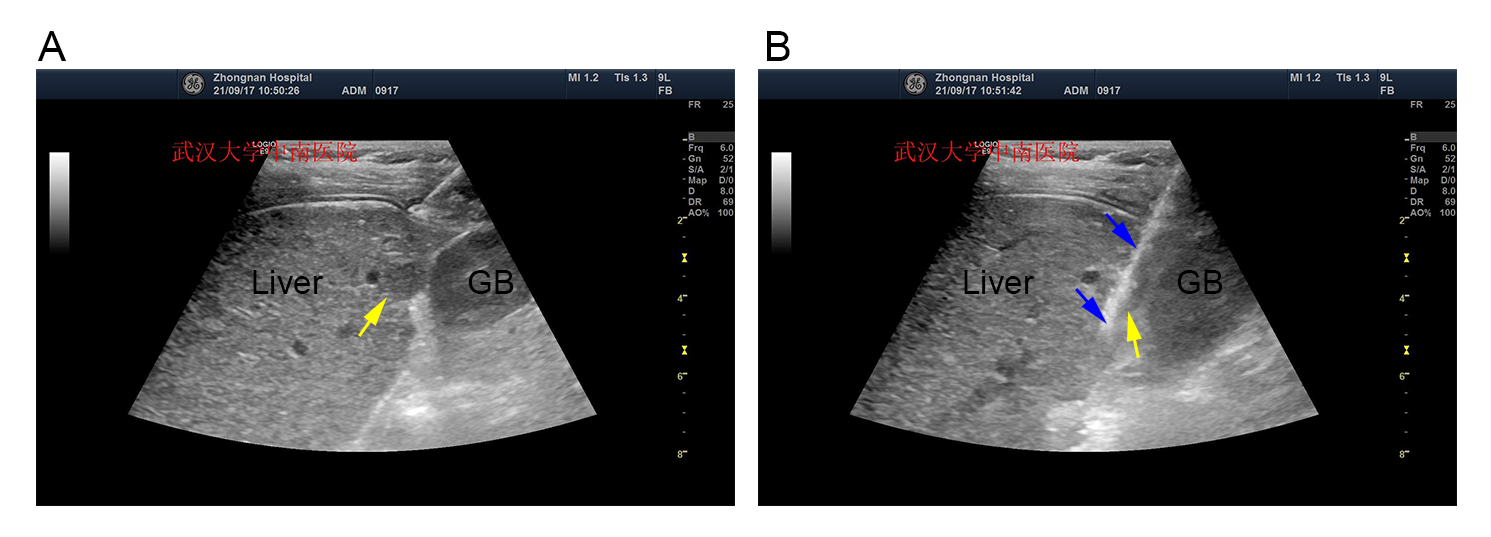

Supplement: Supplementary file 2 [file medi-103-e40018-s002.tif]
